# Epidemiological and Genomic analysis of *Vibrio parahaemolyticus* isolated from imported travelers at the port of Shanghai, China (2017-2019)

**DOI:** 10.1186/s12866-024-03303-7

**Published:** 2024-04-26

**Authors:** Danlei Liu, Lei Zhou, Zilei Zhang, Ying Zhang, Zhiyi Wang, Shenwei Li, Yongqiang Zhu, Huajun Zheng, Zilong Zhang, Zhengan Tian

**Affiliations:** 1 Shanghai International Travel Healthcare Center, Shanghai Customs District P. R. China, Shanghai, 200136 China; 2 Shanghai-MOST Key Laboratory of Health and Disease Genomics, NHC Key Lab of Reproduction Regulation, Shanghai Institute for Biomedical and Pharmaceutical Technologies, School of Life Sciences, Fudan University, Shanghai, 200237 China; 3 Inspection and Quarantine Technology Communication Department, Shanghai Customs College, Shanghai, 200136 China; 4 State Key Laboratory of Applied Microbiology Southern China, Guangdong Provincial Key Laboratory of Microbial Culture Collection and Application, Guangdong Open Laboratory of Applied Microbiology, Guangdong Institute of Microbiology, Guangzhou, 510070 China

**Keywords:** Port surveillance, *Vibrio parahaemolyticus*, Whole genome sequencing, Virulence factor, Antibiotic resistance

## Abstract

**Background:**

*Vibrio parahaemolyticus* is the predominant etiological agent of seafood-associated foodborne illnesses on a global scale. It is essential to elucidate the mechanisms by which this pathogen disseminates. Given the existing research predominantly concentrates on localized outbreaks, there is a pressing necessity for a comprehensive investigation to capture strains of *V. parahaemolyticus* cross borders.

**Results:**

This study examined the frequency and genetic attributes of imported *V. parahaemolyticus* strains among travelers entering Shanghai Port, China, between 2017 and 2019.Through the collection of 21 strains from diverse countries and regions, Southeast Asia was pinpointed as a significant source for the emergence of *V. parahaemolyticus*. Phylogenetic analysis revealed clear delineation between strains originating from human and environmental sources, emphasizing that underlying genome data of foodborne pathogens is essential for environmental monitoring, food safety and early diagnosis of diseases. Furthermore, our study identified the presence of virulence genes (*tdh* and *tlh*) and approximately 120 antibiotic resistance-related genes in the majority of isolates, highlighting their crucial involvement in the pathogenesis of *V. parahaemolyticus*.

**Conclusions:**

This research enhanced our comprehension of the worldwide transmission of *V. parahaemolyticus* and its antimicrobial resistance patterns. The findings have important implications for public health interventions and antimicrobial stewardship strategies, underscoring the necessity for epidemiological surveillance of pathogen at international travel hubs.

**Supplementary Information:**

The online version contains supplementary material available at 10.1186/s12866-024-03303-7.

## Background

*Vibrio parahaemolyticus*, a Gram-negative bacterium with halophilic characteristics, inhabits in coastal estuarine ecosystems and stands as a significant worldwide pathogen accountable for foodborne infections [1, 2]. The escalating occurrence of outbreaks of *V. parahaemolyticus* are closely tied to climate change, posing a significant public health risk [3]. Investigating the potential transmission pathways of *V. parahaemolyticus*, from its environmental reservoir to human exposure through retail channels, emerges as an imperative stride in mitigating the global prevalence of acute gastroenteritis.

Nations across Asia have reported significant impacts of *Vibrio* infections on human health, thereby detrimentally affecting the seafood industry [4]. To facilitate research in this field, the Foodborne *Vibrio parahaemolyticus* Genome Database (FVPGD) has been established, encompassing a compilation of 643 *V. parahaemolyticus* strains obtained from 39 cities in China [5]. Moreover, from 2013 to 2017, there was a comprehensive examination in southeastern China where 1,220 *V. parahaemolyticus* strains were isolated and analyzed [6]. The escalating incidence of *V. parahaemolyticus* infections in China highlights the exigent requirement to assess the prevalence and genetic heterogeneity of this virulent bacterium [5].

The global spread of *V. parahaemolyticus* underscores the imperative to enhance our comprehension of its heightened propagation. As most of the previous studies were conducted independently and focused on local outbreaks, there is a need to perform comprehensive investigations to capture cross-border strains of *V. parahaemolyticus* at the whole-genome level. As the most densely populated urban center in China, Shanghai is witnessing an escalating connectivity within the global city network [7]. Notably, the city boasts two commercial airports, Shanghai Pudong International Airport and Shanghai Hongqiao International Airport, rendering it the world’s fifth-busiest gateway. This confluence of factors amplifies Shanghai’s role as an ideal sentinel for monitoring the global transmission dynamics of *V. parahaemolyticus*.

To trace the origin of *V. parahaemolyticus*, serological typing, pulsed-field gel electrophoresis, multilocus sequence typing (MLST), and whole genome sequencing (WGS) have been widely employed [6]. Nowadays, 16 O serotypes and 71 K serotypes can be identified using commercial antisera [8]. Nonetheless, this method is naturally limited by challenges including its costly, intricate protocols, potential for cross-immunoreactivity, and need for subjective interpretation [9]. WGS of *V. parahaemolyticus* isolates and comparative genomics analyses have revealed various mutations, chromosomal rearrangements and gene gain or loss events caused by duplication or horizontal gene transfer [10]. Bioinformatics methods such as the VPsero [11] and Kaptive database [12] have been developed to identify gene clusters associated with O-loci (lipopolysaccharide, LPS) and K-loci (capsular polysaccharide, CPS) for serotype determination of *V. parahaemolyticus*. Clinical isolates of *V. parahaemolyticus* also contain numerous virulence factors, including the thermostable direct hemolysin (*tdh*) and the *tdh*-related hemolysin (*trh*) [1, 13].

Our research conducted a pangenome analysis to genetically characterize isolates of 21 *V. parahaemolyticus* strains that were collected from imported travelers in Shanghai port, China during 2017-2019. Furthermore, this study enhanced our comprehension of the worldwide spread of *V. parahaemolyticus* and the genetic factors that affect its resistance to antimicrobial substances. Results had implications for public health interventions and strategies to preserve the effectiveness of antimicrobial drugs, highlighting the importance of ongoing monitoring and control of this pathogen.

## Methods

### Sample collection

Shanghai Customs adopted the principle of voluntary reporting and sample collection paradigm to facilitate its disease surveillance efforts. In instances where diarrhea-like symptoms were suspected among inbound travelers, a proactive approach was adopted whereby passengers were requested to self-disclose any abnormal health manifestations. Following the attainment of passengers’ informed consent, the process entailed the collection of anal swab samples by medical personnel stationed at the airport’s customs facility [14, 15]. Subsequently, these samples were meticulously stored under refrigeration conditions and expeditiously conveyed to the Shanghai International Travel Healthcare Center for comprehensive testing and characterization.

### Pathogen detection and isolation of *V. parahaemolyticus*

DNA/RNA of swab samples was extracted using a magnetic beads nucleic acid isolation kit (BioPerfectus technologies, China, Catalog No. SDK60104) following the manufacturer’s instructions. The presence of foodborne pathogens including *Salmonella* spp., *Shigella* spp., *Vibrio* cholerae, *Vibrio* parahaemolyticus, *Escherichia* coli O157:H7, Rotavirus A and GI/GII Norovirus were detected using commercial qPCR kit (Bioperfectus Technologies, Shanghai, China) according to the voluntary border screening surveillance strategy of entry points. The qPCR was performed using the QuantStudio 5 Real Time PCR system (Thermo Fisher, USA) and data were analyzed using the QuantStudio Design and Analysis software. In adherence to the established criterion, samples recording a Ct value below 35 were categorized as positive outcomes for *V. parahaemolyticus* detection.

*V. parahaemolyticus* strains were isolated according to the GB 4789.7-2013 food microbiological examination (National Food Safety Standards of China) [16]. Samples were streaked onto thiosulfate citrate bile-salt sucrose (TCBS) agar plates and incubated at 37 °C for 18-24 h. Presumptive colonies (green or blue green colonies, 2-3 mm in diameter) were then transferred to Chromogenic Vibrio Medium and incubated at 37 °C for 24 h. Genomic DNA was extracted using Bacteria Genomic DNA Extraction kit with magnetic beads (Bioperfectus technologies, Catalog No. SDK60108). Species identification was further confirmed through 16s rRNA gene sequencing. All positive *V. parahaemolyticus* colonies were preserved in Brucella broth with 17.5% glycerol and stored in Biological Sample Bank of Shanghai International Travel Healthcare Center.

### DNA extraction and shotgun genome sequencing

All isolates were cultivated in tryptic soy broth medium (HuanKai Microbial, China) overnight at 37 °C. Genomic DNA was extracted using Bacteria Genomic DNA Extraction kit with magnetic beads (Bioperfectus technologies, Catalog No. SDK60108). Subsequently, DNA integrity and quantity were assessed on a Nanodrop UV-vis spectrophotometer (Thermo Fisher, USA) and 1% agarose gel electrophoresis visualized with ethidium bromide in a ChemiDoc XRS photodocumenter (BioRad, USA). Paired-end libraries were constructed using the TrueSeq DNA Sample Prep Kit (Illumina, USA) according to the manufacturer’s instructions. The libraries were subjected to purification using AMPure XP Beads (Beckman Coulter, USA). For the sequencing phase, the Illumina X-10 platform was employed, generating 2 × 150 bp paired-end reads, consequently accumulating a total of 2 GB data per individual sample.

### De Novo assembly and annotation

Raw reads were trimmed using Trimmomatic v0.36 [17]. The trimmed reads were subjected to assembly using Velvet v1.2.03. Predictions of putative open reading frames (ORFs) within each strain were accomplished using Glimmer3 [18]. Subsequently, the functionality of these ORFs was inferred using BLASTP against the NR protein database of the National Center for Biotechnology Information (NCBI) [19]. To further enhance the comprehension of the gene functions, a multifaceted annotation process was undertaken including pathway annotation and COG functional annotation, all of which contributed to unraveling the intricate functional attributes embedded within the genes of interest. To identify the conservation of *V. parahaemolyticus* strains, average nucleotide identity (ANI) through alignment-free approximate sequence mapping was calculated using Fast Average Nucleotide Identity (FastANI) [20]. Reads were aligned against the* V. parahaemolyticus* reference genome, RIMD 2210633 (assembly GCF_000196095.1).

The prokaryotic Pan-Genome Analysis Pipeline (PGAP) was implemented to perform a pan-genome analysis of 21 *V. parahaemolyticus* genomes with a threshold of 95% identity and coverage [21]. Antimicrobial resistance (AMR) genes were identified using the Comprehensive Antibiotic Resistance Database (CARD), and virulence genes were identified using the virulence finder database (VFDB) [22], with a stringent threshold of 1e-50.

### Serotyping and phylogenetic analysis

Comparison of the serotype-specific genes in VPsero database [9] with 21 human-derived *V. parahaemolyticus* genomes was performed by BLASTN to identify potential serotypes. Meanwhile, the 21 genomes were uploaded to the Kaptive website (https://kaptive-web.erc.monash.edu/) for review and supplementation [10], aiming to obtain a more comprehensive serotype classification. In Kaptive database, the nomenclature for the O- and K- loci was adopted based on the *Klebsiella* capsule synthesis loci, each specific O- and K- locus was separately denoted as OL (O-locus) and KL (K-locus), followed by a unique numerical identifier.

To investigate the potential avenues of cross-transmission of *V. parahaemolyticus*, 74 environmental or foodborne genomes from diverse countries and collection dates were selected and downloaded from NCBI (https://www.ncbi.nlm.nih.gov/assembly/?term=vibrio). A phylogenetic tree of the 21 human-derived and 74 environmental or foodborne *V. parahaemolyticus* isolate was built using IQ-TREE based on the concatenation of the core genomes [23]. The evolutionary distances were computed using the Maximum Likelihood method. The phylogenetic tree was visualized and annotated using Interactive tree of Life (iTOL) [24].

## Results

### Epidemiological features of *V. parahaemolyticus* isolates

**Fig. 1:**
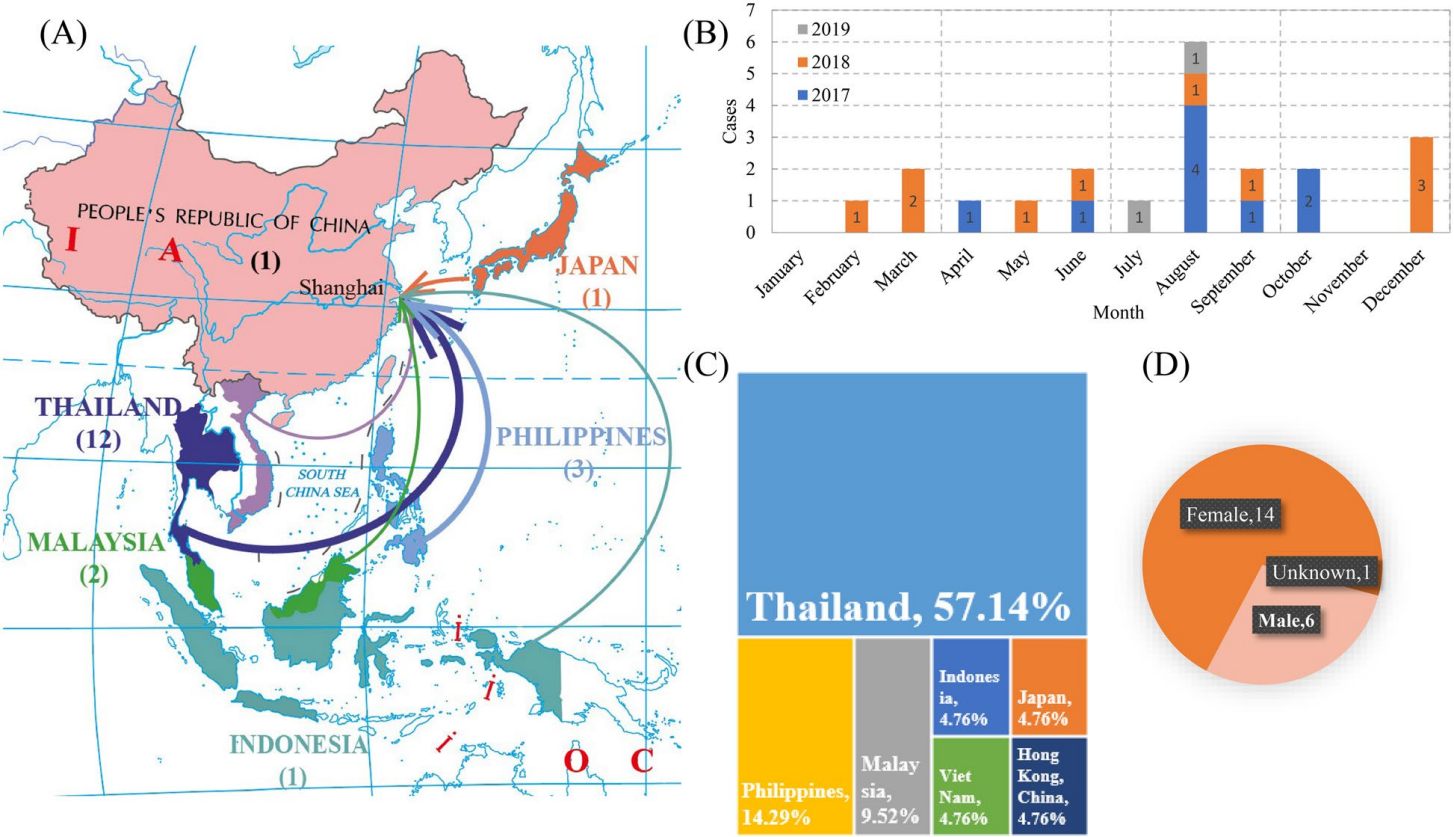
The information of epidemiological and genomic features of 21 human-origin *V. parahaemolyticus* isolates. **A** The regional distribution of the strains. Arrows in different colors represent diverse countries where samples isolated from. The width of arrows reflects the number of the strains. **B** The number of strains isolated in different months from 2017 to 2019. Different years are marked in diverse colors. **C** The proportion of strains isolated from different countries. The size of the rectangle represents the number of isolates collected in corresponding locations. **D** Percentage of different sample genders is shown by pie chart

In this study, a total of 1,435 anal swab samples were collected from imported travelers ranged from 2017 to 2019, with 35 positive cases being detected, for a positive rate of 2.44% for the *V. parahaemolyticus*. Twenty-one (21/35, 60.0%) human-origin strains were successfully isolated and purified for further analysis (Table 1). According to statistics, these strains were traced back to seven Asian countries and regions (Fig. 1A). Notably, Thailand accounted for the largest proportion of 57.14%, followed by the Philippines accounted for 14.29%, and Malaysia accounted for 9.52% (Fig. 1C). To gain insights into the temporal dynamics of *V. parahaemolyticus* incidents, a comprehensive analysis of the distribution pattern across quarters was undertaken. The distribution of *V. parahaemolyticus* cases exhibited noteworthy variations across quarters. The third quarter stood out with a peak incidence rate of 42.86%, closely followed by the fourth quarter which accounted for 23.81% of the cases. A detailed examination of the data unveiled that the highest surge in cases was recorded during the month of August, accounting for 28.57% of the total cases over the course of three years (Fig. 1B). Upon delving into the demographic profile of the affected individuals, it was ascertained that the mean age of the cases was approximately 33.27 years old. Gender distribution revealed that females constituted a significant majority, comprising 66.67% of the cases (Fig. 1D).

**Table 1:** Detailed information and serotyping analysis of 21 isolates collected in Shanghai Port, China

	Information of epidemiological survey				Result of genome sequencing					ST	VPsero serotype			Kaptive serotype		
ID	Date	Region	Gender	Age	Gene	Protein	RNA	Scaffold	Length (bp)		O-loci	K-loci	Ox:Ky	O- loci	K-loci	OLx:KLy
SHP-1108	8/31/2017	Philippines	Male	24	4,748	4,748	116	17	5,190,976	3	7	68	O7:K68	OL4-1	KL68-1	OL4-1:KL68-1
SHP-1117	10/10/2017	Viet Nam	Female	-	4,581	4,581	119	17	5,078,856	3	3	6	O3:K6	OL3_or_OL13	KL6	OL3_or_OL13:KL6
SHP-1177	10/11/2017	Philippines	Female	-	4,666	4,666	111	33	5,157,286	17	12	4	O12:K4	OL4	KL4	OL4:KL4
SHP-1179	6/20/2017	Thailand	Female	56	4,602	4,602	110	22	5,038,963	2516	4	8	O4:K8	OL4	KL8	OL4:KL8
SHP-1269	8/6/2017	Hong Kong, China	Female	11	4,603	4,603	114	13	5,104,622	3	3	6	O3:K6	OL3_or_OL13	KL6	OL3_or_OL13:KL6
SHP-1361	4/8/2017	Japan	Male	22	4,729	4,729	106	36	5,108,367	-	2	-	-	OL2	KL22	OL2:KL22
SHP-1429	9/13/2017	Thailand	Female	40	4,669	4,669	102	18	5,153,566	-	12	55	O12:K55	OL4	KL55	OL4:KL55
SHP-1434	8/12/2017	Thailand	Female	28	4,625	4,625	119	24	5,075,979	3	3	6	O3:K6	OL3_or_OL13	KL6	OL3_or_OL13:KL6
SHP-1678	8/19/2017	Thailand	-	-	4,652	4,652	121	20	5,127,586	3	3	6	O3:K6	OL3_or_OL13	KL6	OL3_or_OL13:KL6
SHP-1433	2/26/2018	Philippines	Female	34	4,615	4,615	120	21	5,093,560	3	3	6	O3:K6	OL3_or_OL13	KL6	OL3_or_OL13:KL6
SHP-1611	5/21/2018	Thailand	Male	32	4,606	4,606	104	29	4,990,142	635	-	-	-	OL1	KL118	OL1:KL118
SHP-1675	8/12/2018	Thailand	Female	10	4,548	4,548	112	25	4,991,106	-	12	-	-	OL10	KL24	OL10:KL24
SHP-1847	3/17/2018	Thailand	Male	36	4,576	4,576	126	22	5,077,773	3	3	6	O3:K6	OL3_or_OL13	KL6	OL3_or_OL13:KL6
SHP-1926	3/21/2018	Thailand	Male	32	4,688	4,688	122	24	5,128,246	3	3	6	O3:K6	OL3_or_OL13	KL6	OL3_or_OL13:KL6
SHP-1949	6/21/2018	Thailand	Female	44	4,647	4,647	115	15	5,056,207	-	12	9	O12:K9	OL4	KL9	OL4:KL9
SHP-1780	9/14/2018	Malaysia	Female	11	4,763	4,763	115	15	5,190,902	3	4	68	O4:K68	OL4	KL68-1	OL4:KL68-1
SHP-3796	12/23/2018	Thailand	Male	66	4,629	4,629	99	42	5,068,935	199	-	-	-	OL1	KL123	OL1:KL123
SHP-8214	12/19/2018	Malaysia	Female	35	4,604	4,604	98	36	5,015,880	2516	12	8	O12:K8	OL4	KL8	OL4:KL8
SHP-8216	12/19/2018	Indonesia	Female	40	4,614	4,614	95	34	5,021,272	2516	12	8	O12:K8	OL4	KL8	OL4:KL8
SHP-3690	7/4/2019	Thailand	Female	45	4,622	4,622	98	51	5,079,348	-	1	-	-	OL1	KL123	OL1:KL123
SHP-8502	8/14/2019	Thailand	Female	33	4,721	4,721	98	40	5,107,560	2516	12	-	-	OL4	KLUT5	OL4:KLUT5

### Genomic features of 21 human-origin *V. parahaemolyticus* isolates

Genome sequencing of the 21 *V. parahaemolyticus* isolates revealed an average genome size of 5.08 ± 0.058 Mb, with variations spanning from 4.99 Mb (SHP-1611) to 5.19 Mb (SHP-1108). The gene content analysis illustrated that these genomes, in aggregate, consisted of an average of 4,643 protein-encoding genes (Table 1). Evidencing the degree of genomic similarity, the average nucleotide identity (ANI) was evaluated among the 21 isolates and the reference genome. The presence and absence of genes was visualized in the highest and lowest ANI value genomes compared with the rest of isolates, and found that most regions of the strain SHP-1611 with the lowest ANI value (98.30%) are consistent with the reference genome RIMD 221063. The pan-genome of the 21 *V. parahaemolyticus* genomes consisted of 97,508 protein-coding genes, of which, 2,461 core genes were identified. Rarefaction analysis demonstrated an open pan-genome, indicative of the diversity limitation within the gene pool.

### Serotyping analysis and phylogenetic relatedness

Combining the serotype classification from the VPsero database and Kaptive database (Table 1), it was observed that the main serotype of these 21 strains was O3:K6 or OL3:KL6 (7/21, mainly detected in travelers originating in Thailand), consistent with the prevailing trend of *V. parahaemolyticus* in the worldwide. For example, strains SHP-1611 and SHP-3796, which lacked serotype classification in the VPsero database, were categorized as serotype OL1:KL118, while SHP3796 was categorized as OL1:KL123 in the Kaptive database, respectively. Therefore, these results could be a useful basic for clinical treatment and basic research investigation.

**Fig. 2:**
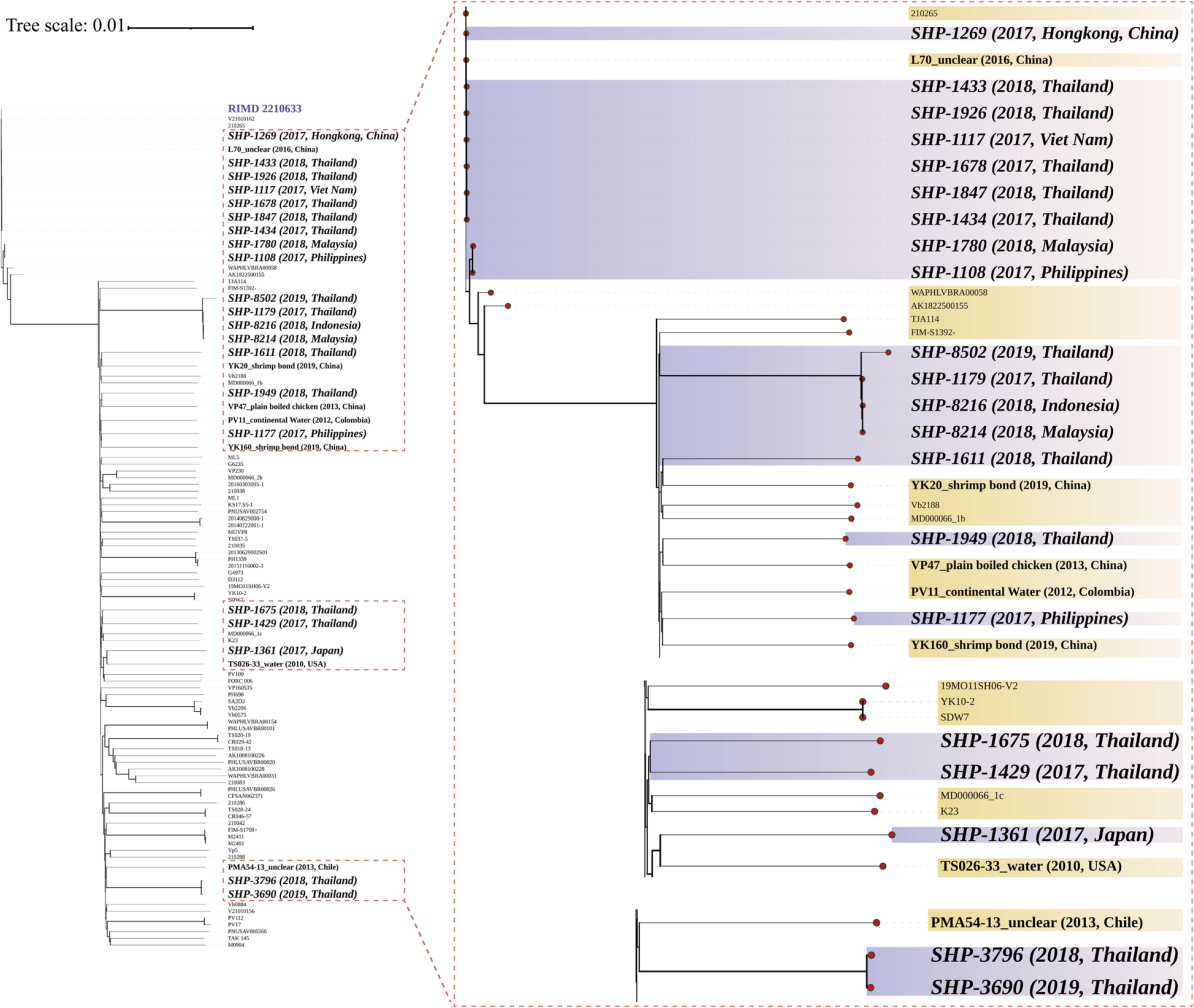
The phylogenetic tree of 96 *V. parahaemolyticus* isolates. The tree was constructed based on the alignment of concatenated core genome of 96 strains, including 21 isolates detected in this study and 74 genomes downloaded from NCBI collected in different years and geographical regions. The reference genome, RIMD 2210633 was also added. The comprehensive evolutionary tree is shown on the left, while the right side provides an enlarged view of the relationship between 21 human-derived *V. parahaemolyticus* isolates (purple strip) and other strains derived from environmental other sources (yellow strip)

Seventy-four strains downloaded from NCBI were distributed in fourteen countries, including China, Mexico, Viet Nam, Colombia, Venezuela, Malaysia, Nigeria, Thailand, Philippines, South Korea, India, Chile, Peru and the USA, with samples from environmental or foodborne sources ranging from 2010 to 2019 (Additional file 1). According to the phylogenetic tree based on 96 concatenated genomes of 1,675 core genes, there was a close genetic relationship between the isolates detected in Thailand, Indonesia, Malaysia and Philippines (e.g., SHP-8502, SHP-8214, SHP-8216 and SHP-1179) (Fig. 2). Strains of Thailand isolated in different years still shared close genetic relationship (e.g., SHP-3690 and SHP-3796, SHP-1675 and SHP-1429). Only three strains separated in Thailand were found to have a close phylogenetic relationship with strains collected from the Chinese environment, such as strains SHP-1611 (2018, Thailand) had a tight phylogenetic connection with YK20 isolated from shrimp bond in 2019, China.

### Distribution of potential virulence-associated genes and antimicrobial resistance genes

**Fig. 3:**
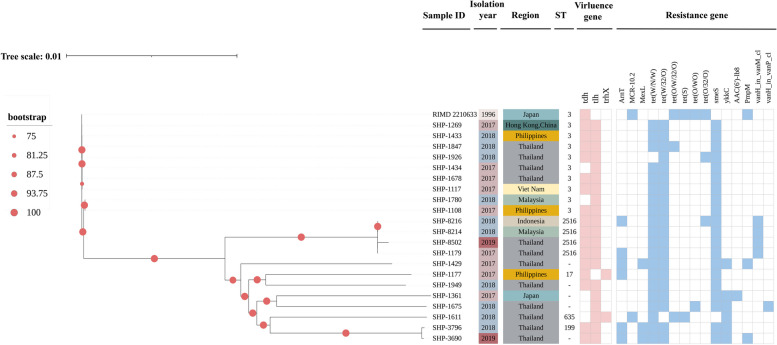
Maximum-likelihood phylogeny using core genome alignment of 21 *V. parahaemolyticus* isolates. Sample ID represents the nomenclature for the strains, Isolation year means the concrete year of isolation, ST describes the sequence types, the presence of the three most common virulence genes in each strain is labeled with red stripe and the blue represents the distribution of resistance genes of the top five antibiotics contained the most predicted resistance genes (peptide, disinfecting agents and antiseptics, tetracycline, aminoglycoside and glycopeptide). The phylogenetic tree was rooted to a reference genome, *V. parahaemolyticus* RIMD 2210633

The exploration of evolutionary relationships was further deepened by conducting a phylogenetic analysis based on the 2,461 core genes encompassing a collection of 22 *V. parahaemolyticus* strains, including reference genome RIMD 2210633 (Fig. 3). Isolates sharing the same Sequence Type (ST) were perceptibly grouped together within the Maximum Likelihood (ML) tree. Nine of the 21 *V. parahaemolyticus* isolates were attributed to ST3, four isolates attributed to ST2516, and three isolates separately assigned to ST17, ST635, and ST199. Five additional isolates remained untyped. It’s noteworthy that despite the variance in both geographic origins and the temporal context of collection, a discernible pattern emerged. Regional clustering was evident, with strains originating from Southeast Asian countries and regions displaying a marked similarity.

Turning attention to potential virulence-associated gene detection, the examination spotlighted 15 virulent mechanisms governed by the predicted genes (Additional file 2). These genes predominantly participated in adhesion, exotoxin production, and effector delivery systems, constituting pivotal virulence attributes. Remarkably, the presence of the *tlh* gene, encoding thermolabile hemolysin, a specific marker of *V. parahaemolyticus*, was identified in 20 out of 21 strains (95.24%). In stark contrast, the occurrence of the *tdh* and *trh* genes was observed in 16 out of 21 strains (76.19%) and two out of 21 strains (9.52%). However, only SHP-3796 contained both the *tdh* and *trh* genes.

Through a comprehensive analysis based on the CARD, a robust assortment of 147 drug resistance genes was identified (Additional file 3). On average, each strain was found to harbor approximately 120 antibiotic resistance-related genes, spanning a spectrum of 25 distinct antibiotics. Remarkably, a substantial proportion of these resistance genes encoded efflux pumps, effectively conferring resistance against an array of antibiotics encompassing the top five predicted antibiotics including peptides, disinfecting agents and antiseptics, tetracycline, aminoglycoside, glycopeptide, and more (Fig. 3). The categories of drug-resistant genes contained in these strains indicated the possibility of the presence multidrug resistance. Of the 21 *V. parahaemolyticus* genomes, 20 (95.24%) strains contained the gene *tet* (*W/N/W*), *tet* (*W/32/O*) and *smeS.* Moreover, within the context of STs, the gene *vanH_in_vanM_cl* was solely identified in strains (SHP-8216, SHP-8214, SHP-8502 and SHP-1179) designated as ST2516. This congruence between the ST types and the associated resistance patterns provided intriguing insights into the genetic determinants influencing antibiotic resistance within the *V. parahaemolyticus* population.

## Discussion

The true global burden of illness caused by *Vibrio* exposure is currently underestimated due to the limited detection and surveillance of vibriosis [11]. This study represents a comprehensive genomic investigation of *V. parahaemolyticus* isolates and provides insight into the diversity and epidemiology of cross-border spread pathogens. The use of WGS provides a high level of resolution, making it crucial for public health and safety to mitigate the risk of cross-border transmission and spread of the pathogen in China. Cross border surveillance is a key issue in understanding their epidemiology and health risks to human [25].

With the increasing accessibility of genome-wide sequencing technologies, numerous pathogen genomes have become available. These genomes have facilitated the identification of effector proteins with diverse functions in various pathogens [26]. The study benefits from the utilization of network techniques and the construction of databases, which overcome the barriers associated with cross-border pathogen surveillance. Additionally, pangenome analyses represent an adequate comparative genomics approach to investigate the intraspecific diversity of *V. parahaemolyticus* by analyzing the core and accessory genomes of different isolates of species.

The findings indicated the utilization of WGS data from foodborne bacteria is crucial for monitoring the environment, ensuring food safety, and diagnosing diseases early. Advances in sequencing technology and reduced costs have contributed to an abundance of bacterial genomic data, enabling a clearer understanding of the transmission patterns of foodborne pathogens.

Travel, including medical tourism, is an important risk factor for the spread of pathogens. Southeast Asia has been identified as a hotspot for the emergence of both extra-intestinal bacteria and gastrointestinal pathogens [27]. Our previous investigation of human norovirus at Shanghai Port has revealed a concentration of cases originating from Southeast Asia, particularly from Thailand [14, 15]. Climate is known to have influence on the distribution of human pathogens [28]. Thailand is predominantly an agricultural country, locates in a tropical zone, which has a hot and humid climate with different epidemiology from other countries [29]. Meanwhile, it is one of the most visited countries globally, improving opportunities for pathogens transmission all around the world. These factors contribute to the formation of a ‘melting pot’ of diverse pathogens. In this study, half of the samples were collected from Thailand (57.14%) during the three years. Therefore, it is essential to intensify *V. parahaemolyticus* surveillance in Thailand and other Southeast Asian countries to detect emerging strains with epidemic potential at an early stage.

For the serotype identification of 21 *V. parahaemolyticus* isolates, the Kaptive database provided a relatively comprehensive serological typing when serotypes cannot be conclusively determined in the VPsero database, such as SHP-1611 previously described (Table 1). But the O-serogroup genetic determinant region (OGD region) in this database, serotype O13 was found to share 100% identity with corresponding region of O3 serotype, for which the whole genome has been published previously [30]. Therefore, in Kaptive O-database, the serotype O3 or O13 genomes were categorized as OL3_or_OL13. For example, in the VPsero database, the serotype of SHP-1611 was identified as O3:K6, but in the Kaptive website, it was identified as OL3_OL13:KL6. Notably, these two classification methods exhibit a high degree of consistency in the analysis of the K antigen.

In terms of pathogen risk assessment, virulence genes (*tdh*, *trh*) were found in most of isolates in this study. The presence of these two virulence genes in *V. parahaemolyticus* is commonly used in clinical, surveillance, and research testing as an indicator of pathogenicity [1, 12]. The *tlh* gene, as the unique marker for *V. parahaemolyticus* identification, revealed in all the 74 isolates obtained from environmental or other sources that were included in our study [31] (Additional file 1). Furthermore, the presence of the *tdh* gene was nearly negligible in environmental strains, while it was extensively found in clinical strains, consistent with findings from prior reports [32]. It is known that horizontal gene transfer plays a role in the transfer of pathogenicity [33]. Therefore, it is important to continue monitoring and investigating other pathogenicity markers in these products.

There is strong evidence to suggest that AMR can disseminate globally across borders [34]. Multi-resistant strains are being distributed worldwide through air travel and other forms of travel across borders [35]. Our AMR isolates demonstrated resistance to three or more antimicrobial agents. This relatively high prevalence of these strains and their AMR profiles reveal the potential public health problems associated with illegal import of food in passengers’ luggage. Therefore, it is imperative for nations to collaborate in order to mitigate the emergence of global AMR. Also, appropriate measures should be taken to prevent the spread of these resistant-bacterial isolates by increasing awareness about health and hygiene and by restricting the random use of antibiotics and antiseptics [36].

## Conclusions

Through the examination of 21 strains sourced from diverse countries and regions, our research has pinpointed Southeast Asia as a significant hotspot for the prevalence of *V. parahaemolyticus*. Furthermore, phylogenetic analysis has delineated clear delineations between strains originating from human and environmental sources, underscoring the restricted transmission dynamics between these two reservoirs. Additionally, the detection of virulence genes and antibiotic resistance-related genes in the majority of isolates underscores their pivotal contributions to the pathogenicity of *V. parahaemolyticus*-induced illnesses. The findings of this research enhanced our comprehension of the worldwide spread of *V. parahaemolyticus* and carried substantial implications for future public health prevention efforts.

### Supplementary Information

**Supplementary Material 1.**

**Supplementary Material 2.**

**Supplementary Material 3.**

## Data Availability

The raw sequencing data reported in this study have been deposited in the Genome Sequence Archive in the National Genomics Data Center, Beijing Institute of Genomics (China National Center for Bioinformation), Chinese Academy of Sciences, under accession number CRA014330 and are publicly accessible at https://bigd.big.ac.cn/gsa.
